# Undifferentiation of Somatic Responses to Emotions in a Case of Functional Amnesia

**DOI:** 10.1155/2008/245495

**Published:** 2008-04-11

**Authors:** E. Tramoni, S. Khalfa, O. Felician, A. Trebuchon-Da Fonseca, M. Poncet, M. Ceccaldi

**Affiliations:** ^1^INSERM U 751MarseilleF-13385France; ^2^Aix-Marseille UniversitéFaculté de MédecineMarseilleF-13385France; ^3^Assistance Publique – Hôpitaux de MarseilleHôpital La TimoneService de Neurologie et NeuropsychologieMarseilleF-13005France; ^4^Assistance Publique – Hôpitaux de MarseilleHôpital La TimoneService de Neurophysiologie CliniqueMarseilleF-13005France

**Keywords:** Retrograde memory, emotional flattening, autonomic nervous system, functional amnesia

## Abstract

The term functional amnesia (FA) has been proposed for cases of memory impairment presenting with severe retrograde amnesia in the absence of cerebral injury or history of psychiatric disturbance. Emotional flattening has often been reported alongside FA, however the mechanism of such a modification is unknown. This study aimed to explore the emotional processing in a rare case of a patient with FA complaining of severe emotional flattening.

We presented ecological dynamic video stimuli conveying strong peaceful and fearful emotions to the patient and 13 controls. We then explored their emotional responses considering both conscious emotional judgements and automatic psychophysiological responses (skin conductance) and facial muscular activity (corrugator supercilii). Both patient P.P. and controls perfectly recognized the emotions conveyed by the films. However, P.P. failed to showan increased skin conductance and corrugator activity as found in controls during fearful film extracts compared with peaceful extracts. Taken together, these finding demonstrate the presence of an emotional deficit, characterized by a failure to generate appropriate somatic responses to positive and negative stimuli. Although this altered somatic processing did not interfere with PP’s explicit recognition of emotion, it modified his emotional experience, thereby constituting a possible explanation for his emotional flattening. This study therefore suggests that FA is not limited to a mnemonic impairment, but is a more complex disorder, involving also the processing of emotionally loaded experiences.

## 1. Introduction

Retrograde amnesia is characterized by complete or partial loss of episodic and/or semantic information acquired before a critical incident, and is usually accompanied by anterograde amnesia. However, focal retrograde amnesia, with preservation of anterograde memory, has been reported in several case reports [17–19, 41]. Aetiology independent, focal retrograde amnesia may be associated with major cerebral pathology or may occur without any overt structural lesions as a consequence of severe psychological trauma or minor concussion. The term "functional amnesia" (FA) [6] has been proposed for cases presenting with symptoms of retrograde amnesia with no evidence of structural brain damage or history of psychiatric disturbance.

Patients with FA frequently show an emotional flattening [28,29], with a lack of emotional colorization in the retrieval of personal or non-personal newly acquired information, and often a loss of emotional ex-

ISSN 0953-4180/08/$17.00 © 2008 – IOS Press and the authors. All rights reserved perience [3,20,28]. However, to our knowledge, study of emotional processing has never been conducted in detail in subject with FA. This study therefore aimed to explore the emotional processing in a patient with FA complaining of severe emotional flattening. One reliable way of exploring emotional responses is to consider both conscious verbally mediated emotional judgment and implicit somatic emotional reactions. Somatic reactions, studied by psychophysiological parameter registration, have often been demonstrated as distinguishing among basic emotions [4,9,26,35]. Skin conductance change, an index of autonomic arousal, is the most widely used objective measure to access emotional status [22,31,37]. As the face is the locus of a great spectrum of emotional expression, electromyographic measures of activity over *corrugator supercilii* are also used to study negative affects [25]. We hypothesized a possible link between the persistent emotional loss described by the patient and both an altered ability to explicitly recognize emotions and undifferentiated somatic responses to basic emotions.

## 2. Material and methods

### 2.1. Case report

P.P., a 34 year-old right-handed man and tutor of handicapped adults, was admitted because of a sudden and persistent severe retrograde amnesia following a fall off a ladder. He could not remember any personal past events experienced before the onset of his amnesia. On admission, the patient presented with normal neurological status and psychiatric examinations. There was no evidence of dysautonomic failure. Standard imaging (CT scan and MRI) showed no focal cerebral lesion. Neuropsychological standard evaluation including anterograde memory, executive functioning, language, praxis and visuoperceptive skills was normal. He had a global IQ of 115 [39] and a general delayed memory score of 159 [40].

Assessment of autobiographical memory [24] showed an inability to recall any specific events at periods throughout life except for the months following his episode of amnesia (Fig. 1). In addition, P.P.'s most persistent complaint concerned the loss of emotion. He reported that he could neither feel sadness, nor joy and that other persons' emotional states had lost importance for him. For instance, he described an absence of emotion following the tsunami incident in December 2004. Although aware of the victims' sadness he felt no empathy for them. In spite of this complaint, the patient showed a preserved categorization of facial emotional expression [10].

A score of 13 out of 21 in the Hamilton Depression Rating Scale [16] was suggestive of mild depression, however no mood disorder or sad affect was apparent in clinical setting. During the period of evaluation, P.P. was free of medication.

### 2.2. Protocol

Ecological audio-dynamic video stimuli conveying strong emotions were used in a classical passive observation paradigm. Twenty-four commercial film excerpts accompanied with music (12 fearful and 12 peaceful) of 15 to 20 sec duration [21] were presented to the patient and 13 controls (8 women, 5 men; age: $24.6 \pm 8.1$ years) in a pseudo-randomized order.

In order to verify the emotional recognition, participants had to verbally judge if the excerpt was fearful or peaceful following each stimulus presentation.

We recorded sympathetic activity (skin conductance of the left hand) and facial expression (right *corrugator supercilii* muscular activity) during the experiment according to the recommendations of Fowles and coll [11] and Fridlund and Cacioppo [12]. We acquired muscular activity at 1000 Hz and skin conductance at 125 Hz using the Biopac MP system and Acknowledge software 3.6.7. Skin conductance was smoothed at 0.5 sec with a biopac function allowing the calculation of the skin conductance level (SCL). The biopac software also allowed a calculation of the root mean square of the *corrugator*. Before the experiment, we verified all autonomic responses, i.e., that P.P. and each control presented skin conductance responses during inspiration and sudden noise.

For each subject, we subtracted the mean values obtained during the baseline periods (5 sec duration) preceding the stimuli, from the mean values obtained during the first 15 sec of the stimuli and the 5 sec after. These were then averaged according to stimulus categories (fearful or peaceful). Data analyses were then performed on the different values obtained to determine which psychophysiological parameters differed between peaceful and fearful movies.

## 3. Results

Both controls and P.P. recognized perfectly the intended emotion of fear and peacefulness (P.P. score: 100%, control scores: $95.83 +/- 8.5$).

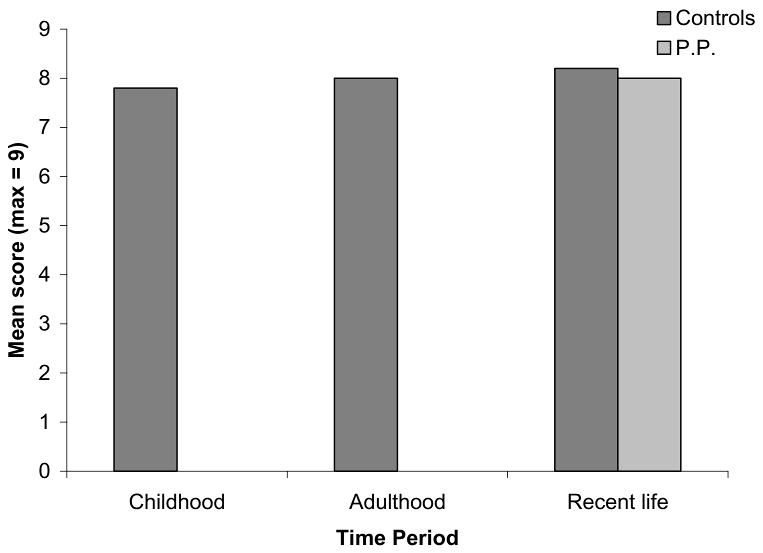

Fig. 1. P.P.'s performance on the Episodic Autobiographical Memory section of the Kopelman Autobiographical Memory Interview (AMI, Kopelman [24]). He obtained pathological concerning performance (= 0) all periods preceding the onset of his amnesia. Concerning more recent life events, he obtained normal scores and could recall detailed personal events experienced between August 2004 (the onset of his amnesia) and March 2005 (date of evaluation).

Psychophysiological results are characterized by extreme values spread out over a wide range and positively skewed. We therefore performed statistical analyses using non-parametric tests. In controls, we found an increased of SCL (U $=$ 9559.5, N $=$ 298, $P < 0.05$) (Fig. 2a) and *corrugator* activity (U $=$ 9271.5, N $=$ 298, $P < 0.05$) (Fig. 2b) during fearful film extracts compared with peaceful extracts. In contrast, in PP, we found no significant difference between peaceful and fearful extracts neither for SCL (U $=$ 64, N $=$ 24, $P = 0.67$) nor for *corrugator* activity (U $=$ 44, N $=$ 24, $P = 0.11$). A data analysis of each control showed that they all exhibited significant differentiation between the two emotions for at least one of the two psychophysiological measures. Finally, even if controls and PP also seem to be differentiated by their SCL in response to fearful films, whereas their *corrugator* activity seems to differ in response to peaceful films, no significant difference was found that may suggest such a dissociation.

These results indicate that despite recognizing the emotions conveyed by the films and unlike the controls, the psychophysiological responses of P.P. did not allow differentiating between peaceful and fearful emotion.

## 4. Discussion

In this case, we found evidence of a dysregulation of emotional processing: P.P. experienced general flattening of emotions, consistent with previous reports of similar cases of FA [3,28,29]. Our data show an emotional flattening caused by neither an inability to explicitly identify emotional situations, nor a general autonomic dysfunction. The major difference between P.P. and the controls concerns the failure of his somatic system to generate distinct patterns of SCL and *corrugator* activity in response to peaceful and fearful films. Then, the psychophysiological study carried out in PP showed a dissociation between preserved cognitive processing of emotion and altered experienced somatic arousal. This is the opposite pattern of the dissociation reported in patients who exhibit preserved physiological response in the absence of verbal report of emotion [2,32,33]. This double dissociation suggests that verbal labels representing emotional features on the one hand, and emotional experiences on the other hand, are subserved by distinct neural systems, as previously proposed [5].

One could claim that P.P. simulated his emotional disorder in trying to avoid feelings during film viewing. However, Gross [15] found that participants who viewed a disgust-eliciting film while suppressing their emotional experience increased their skin conductance relative to those who naturally watched the film. Another hypothesis could be that emotional flattening is generated by a mild depression secondary to the amnesia. However, in P.P., we didn't find reduced electrodermal activity as it has been reported in depression [38]

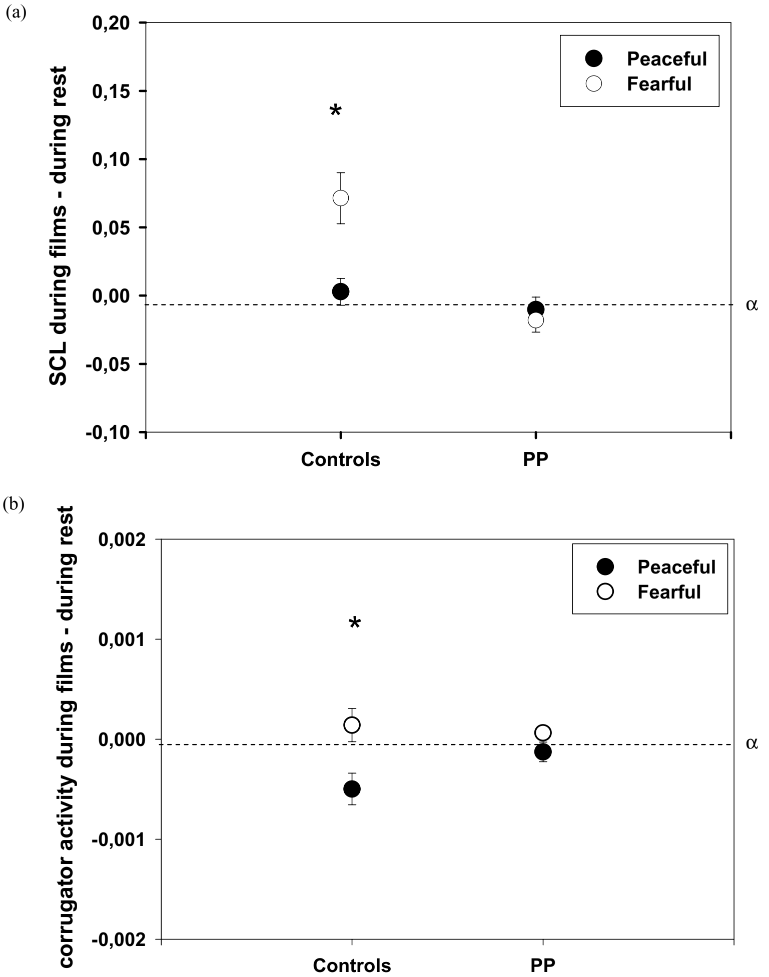

Fig. 2. Psychophysiological responses of patient and controls during peaceful and fearful film extracts viewing. P.P. failed to show an increase in: (a) skin conductance and (b) corrugator activity as found in controls for fearful films in comparison with peaceful films.

and psychiatric disorders [34]. Thus, as the emotional disorder seems neither self-simulated nor caused by depression, we hypothesize that a somatic inability to differentiate between positively and negatively valenced external stimuli constitutes a possible explanation for the emotional flattening. Since this autonomic arousal differentiation involves several cerebral structures like the cerebellum [1], medial temporal structures [13], thalamic/hypothalamic area, midbrain and left lateral prefrontal cortex [27], we cannot definitively exclude the presence of a cerebral lesion non visible with standard imaging methods.

In addition, while we brought evidence for emotional disorder in FA, the putative relationship between these two deficits remains unclear. Kopelman [23] proposed a model postulating that severe stress can sometimes affect frontal/executive systems and thereby inhibit the retrieval of autobiographical memories and additionally knowledge of self and identity. This situation would involve a negative feedback and a severe dampening of the current emotional state resulting in the subject appearing emotionally "flat". Alternatively, altered emotional processing could be responsible for impaired retrieval of personal memories. Yet, recent neuroimaging studies have showed that activity in the hippocampus and cerebral structures involved in emotion are correlated during recollection of personal past events [7,8, 14]. Moreover, Schaefer and Philippot [36] found a positive relation between emotional state at retrieval and the level of phenomenal details of retrieved memories. Then, impaired recollection of past experiences could be related to an inability to arouse the somatic responses associated with the emotional content of these episodes. Further studies should be carried out to explore this relationship between FA and emotional processing.

## Acknowledgements

We are grateful to Doctor Barbeau for helpful comments and advice, to Emily Witty for the revision of the English version of this paper and helpful comments, to Doctor Wicker and Doctor Schon for lending us the stimuli and to the patient and his wife for their implication in this study.
